# Pathological complete response of HER2-positive breast cancer to trastuzumab and chemotherapy can be predicted by HSD17B4 methylation

**DOI:** 10.18632/oncotarget.15118

**Published:** 2017-02-06

**Authors:** Satoshi Fujii, Satoshi Yamashita, Takeshi Yamaguchi, Masato Takahashi, Yasuo Hozumi, Toshikazu Ushijima, Hirofumi Mukai

**Affiliations:** ^1^ Division of Pathology, Exploratory Oncology Research and Clinical Trial Center, National Cancer Center, Kashiwa, Chiba 277-8577, Japan; ^2^ Division of Epigenomics, National Cancer Center Research Institute, Chuo-ku, Tokyo, 104-0045, Japan; ^3^ Department of Medical Oncology, Musashino Red Cross Hospital, Musashino, Tokyo 180-8610, Japan; ^4^ Department of Breast Surgery, Hokkaido Cancer Center, National Hospital Organization, Shiroishi-Ku, Sapporo, 003-0806, Japan; ^5^ Department of Breast and Endocrine Surgery, Ibaraki Clinical Education and Training Center, Faculty of Medicine, Tsukuba University/Department of Breast Surgery, Ibaraki Prefectural Central Hospital, Kasama, Ibaraki 309-1793, Japan; ^6^ Department of Breast and Medical Oncology, National Cancer Center Hospital East, Kashiwa, Chiba 277-8577, Japan

**Keywords:** DNA methylation, HER2-directed therapy, biomarker, breast cancer

## Abstract

Human epidermal growth factor (HER) 2-directed therapy is the standard treatment for HER2-positive breast cancer. Patients who achieved a pathological complete response (pCR) to the therapy are associated with excellent disease-free survival. However, few molecular markers are available to predict pCR. Here, we aimed to establish a DNA methylation marker to predict the response to trastuzumab and chemotherapy. A total of 67 patients were divided into screening (*n* = 21) and validation (*n* = 46) sets. Genome-wide DNA methylation analysis of the screening set identified eight genomic regions specifically methylated in patients with pCR. Among these, HSD17B4 encoding type 4 17β-hydroxysteroid dehydrogenase was most significantly differentially methylated. The differential methylation was confirmed by pyrosequencing (*P* = 0.03), and a cutoff value was determined. This association was successfully validated in the validation set (*P* < 0.001), and patients with pCR were predicted with a high specificity (79%). Multivariate analysis, including tumor stage and hormone receptor status, showed that HSD17B4 methylation was an independent predictive factor (odds ratio: 10.0, 95% confidence interval 2.54–39.50, *P* = 0.001). Combination with ER status and *HSD17B4* methylation improved the specificity up to 91%. Identification of HER2-positive breast cancer patients who would achieve pCR only by trastuzumab and chemotherapy may lead to surgery-free treatment for this group of breast cancer patients.

## INTRODUCTION

Breast cancers with overexpression of the human epidermal growth factor 2 (HER2) receptor tyrosine kinase or amplification of *HER2* are defined as HER2-positive [1, 2]. These constitute approximately 15%–20% of all breast cancers, and used to be characterized by an aggressive clinical course with a propensity for distant metastases within 5 years of diagnosis [2, 3]. However, the emergence of trastuzumab as a treatment has dramatically improved the outcomes of patients with all stages of HER2-positive breast cancer [4–7]. The combination of HER2-directed agents, including trastuzumab and pertuzumab, and chemotherapy such as docetaxel and paclitaxel was recently approved for use in preoperative and metastatic HER2-positive breast cancer patients [8, 9]. Moreover, the increased use of neoadjuvant HER2-directed agents with chemotherapy has been documented [10].

Following treatment with HER2-directed agents and chemotherapy, some patients achieve a pathological complete response (pCR), and these patients are known to have very long disease-free survival [10–12]. Currently, pCR can only be established after patients have undergone HER2-directed therapy and surgery, and we cannot predict responders in advance or identify responders without surgery. However, if we could predict responders in advance, this would greatly contribute to patient stratification, or personalized treatment. Therefore, a novel marker needs to be developed to identify HER2-positive breast cancer patients who will show pCR in HER2-directed therapy with a high specificity.

To screen and validate a novel marker, robust genome-wide analysis and a large number of samples with high-quality clinical information are essential. For robust genome-wide analysis, we herein focused on DNA methylation. Compared with gene expression, DNA methylation can be analyzed using DNA, which has several advantages over RNA such as resistance to degradation and easy handling [13–15]. In addition, DNA methylation is stably inherited upon cell division, and is capable of predicting that a gene cannot be expressed even if induced. Further, even in specimens with contaminating cells, the DNA methylation level can be corrected using the ratio of cancer cells to total cells. This ratio can be obtained by microscopic examination of thin sections stained with hematoxylin and eosin (H&E) from paraffin-embedded tissue specimens or using DNA methylation markers [16].

In the present study, we used genome-wide methylation analysis to identify genomic regions whose DNA methylation status was associated with pCR to trastuzumab and chemotherapy. In this way, surgery-free treatment may become possible for such patients.

## RESULTS

### Genome-wide screening of regions with methylation statuses associated with pCR

To identify genomic regions specifically methylated or unmethylated in the initial biopsy specimens of responders (patients with pCR), genome-wide DNA methylation analysis was conducted using samples in the screening set (Table 1), namely: i) 10 samples from responders (pCR samples), ii) 11 samples from non-responders (non-pCR samples), iii) normal breast epithelial cell lines, and iv) an HER2-positive breast cancer cell line.

**Table 1:** Clinicopathological characteristics of HER2-positive breast cancer patients in the screening and validation sets

	Screening set *N* = 21		Validation set *N* = 46	
	pCR	Non-pCR	pCR	Non-pCR
No. of patients	10	11	12	34
Clinical tumor stage (%)				
cT1	0 (0.0)	2 (18.2)	1 (8.3)	1 (2.9)
cT2	8 (80.0)	8 (72.7)	9 (75.0)	24 (70.6)
cT3	1 (10.0)	1 (9.1)	2 (16.7)	7 (20.6)
cT4	1 (10.0)	0 (0)	0 (0.0)	2 (5.9)
Tumor size (cm)	2–6.5 (median: 3.5)	0–7.0 (median: 3.5)	1.5–7.0 (median: 3.0)	1.4–10.0 (median: 3.5)
Clinical nodal stage (%)				
cN0	3 (30.0)	3 (27.3)	3 (25.0)	16 (47.1)
cN1	4 (40.0)	6 (54.5)	8 (66.7)	15 (44.1)
cN2	1 (10.0)	1 (9.1)	0 (0.0)	3 (8.8)
cN3	2 (20.0)	1 (9.1)	1 (8.3)	0 (0.0)
Clinical stage (%)				
cIIA	3 (30.0)	5 (45.5)	4 (33.3)	15 (44.1)
cIIB	3 (30.0)	3 (27.3)	6 (50.0)	10 (29.4)
cIIIA	1 (10.0)	2 (18.1)	1 (8.3)	7 (20.6)
cIIIB	1 (10.0)	0 (0.0)	0 (0.0)	2 (5.9)
cIIIC	2 (20.0)	1 (9.1)	1 (8.3)	0 (0.0)
Histology (%)				
Invasive ductal carcinoma	9 (90.0)	10 (90.9)	12 (100.0)	33 (97.1)
Invasive lobular carcinoma	0 (0.0)	1 (9.1)	0 (0.0)	1 (2.9)
Others	1 (10.0)	0 (0.0)	0 (0.0)	0 (0.0)
ER^a^ status				
Positive	2 (20.0)	8 (72.7)	3 (25.0)	21 (61.8)
Negative	8 (80.0)	3 (27.3)	9 (75.0)	13 (38.2)
PgR^b^ status				
Positive	3 (30.0)	6 (54.5)	1 (8.3)	16 (47.1)
Negative	7 (70.0)	5 (45.5)	11 (91.7)	18 (52.9)
Ki-67 labelling index (%)	30–60 (median: 45)	20–80 (median: 40)	26.5–80 (median: 60)	10–96.5 (median: 49.3)

^a^Estrogen receptor, ^b^Progesterone receptor.

**Figure 1:**
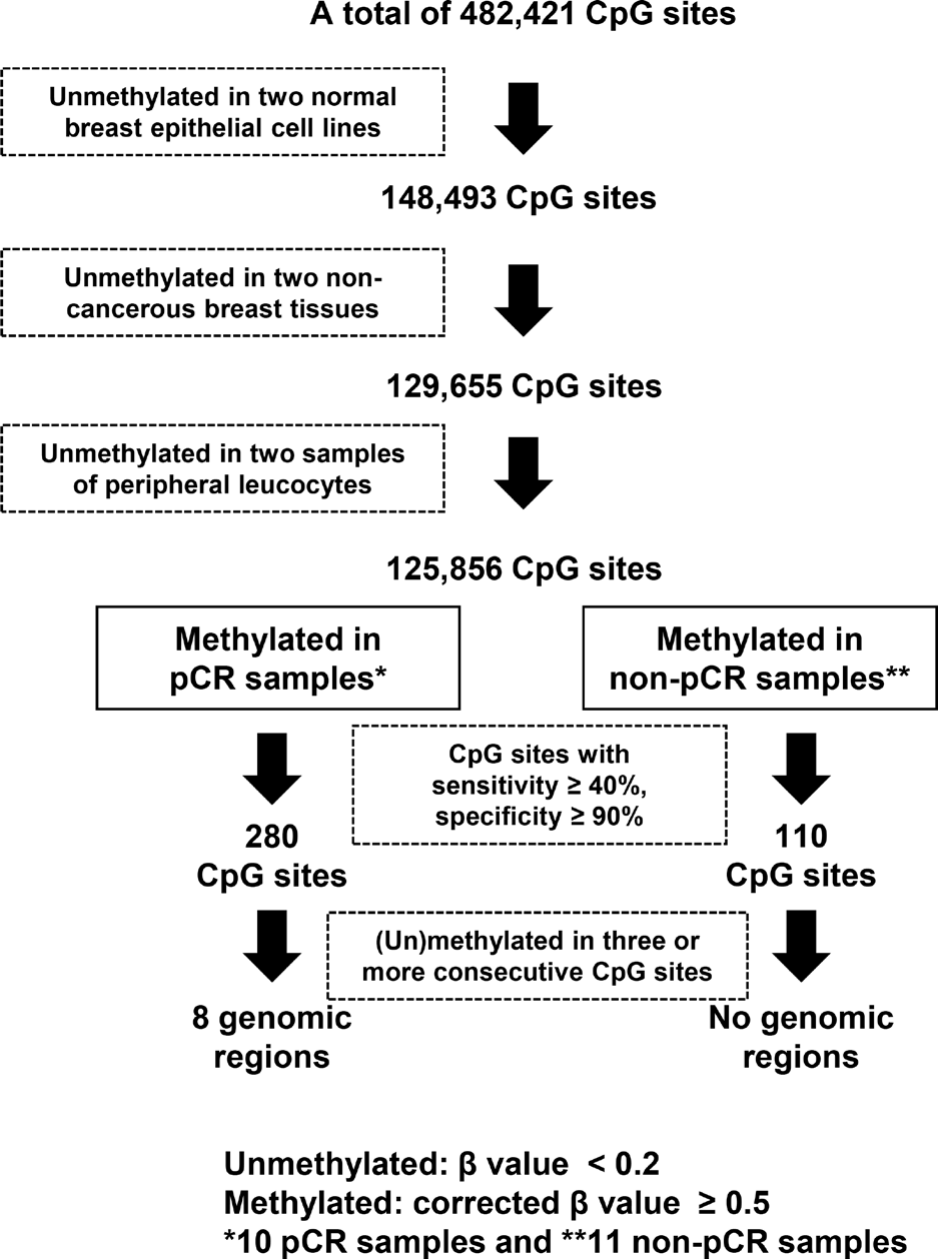
Screening process of genomic regions whose methylation statuses were associated with pCR to trastuzumab and chemotherapy From the 482,421 CpG sites on the Infinium HumanMethylation450 BeadChip array, those unmethylated in normal breast epithelial cell lines, non-cancerous breast tissues, and peripheral leucocytes were selected. A total of 280 CpG sites were preferentially methylated in responders, and 110 CpG sites were in non-responders. By selecting genomic regions with three or more consecutive differentially methylated CpG sites, eight genomic regions were found to be preferentially methylated in pCR samples. A methylated CpG site was defined when its corrected β value was ≥ 0.5. An unmethylated CpG site was defined when its β value was < 0.2.

Among the 482,421 CpG sites, 125,856 were found to be unmethylated (β value < 0.2) in two normal breast epithelial cell lines, two non-cancerous breast tissues, and two samples of peripheral leucocytes (Figure 1). Among these CpG sites, 280 were methylated [corrected β value ≥ 0.5] in four or more of the 10 pCR samples and unmethylated in 10 or more of the 11 non-pCR samples. Conversely, 110 CpG sites were methylated in five or more of the 11 non-pCR samples and unmethylated in nine or more of the 10 pCR samples. Finally, by selecting genomic regions containing three or more consecutive CpG sites that were consistently methylated or unmethylated, we isolated eight candidate genomic regions specifically methylated in the pCR samples (Table 2). No genomic regions were specifically methylated in the non-pCR samples.

**Table 2:** Candidate genomic regions identified by genome-wide DNA methylation analysis

Illumina target ID	Location		Gene symbol	UCSC RefGene_Group	Relation_to_UCSC_CpG_Island	Methylation levels		Incidence				Performance as pCR marker		Pyrosequencing	
					(average beta value ± SD)										
	CHR	MAPINFO				pCR	non-pCR	pCR	non-pCR	pCR	non-pCR	Sensitivity	Specificity	Screening	Validation
cg16689634	1	47489539	*CYP4X1*	1stExon	Island	0.24 ± 0.13	0.14 ± 0.12	4	1	6	9	0.40	0.91	ND	ND
cg24531022	5	102201825	*PAM*	5′UTR;1stExon	Island	0.24 ± 0.15	0.11 ± 0.07	4	0	5	10	0.40	1.00	ND	ND
cg15896301	5	118788229	*HSD17B4*	1stExon;5′UTR	Island	0.29 ± 0.20	0.08 ± 0.12	6	1	4	10	0.60	0.91	Performed	Validated
cg14314653	6	105584709	*BVES*	TSS1500;TSS200	Island	0.31 ± 0.22	0.16 ± 0.12	4	1	6	10	0.40	0.91	ND	ND
cg26252281	6	146864885	*RAB32*	1stExon;5′UTR	Island	0.23 ± 0.11	0.13 ± 0.06	4	1	5	9	0.40	0.91	ND	ND
cg05136452	11	64879891	*TM7SF2*	Body	Island	0.23 ± 0.27	0.08 ± 0.18	4	1	6	10	0.40	0.91	ND	ND
cg08622149	X	3631178	*PRKX*	1stExon	Island	0.22 ± 0.18	0.12 ± 0.12	5	1	5	8	0.50	0.91	ND	ND
cg16978043	X	16888606	*RBBP7*	TSS200	Island	0.23 ± 0.16	0.09 ± 0.11	5	1	4	10	0.50	0.91	Impossible to make primer set	ND

### Confirmation of the methylation status of a candidate genomic region

**Figure 2:**
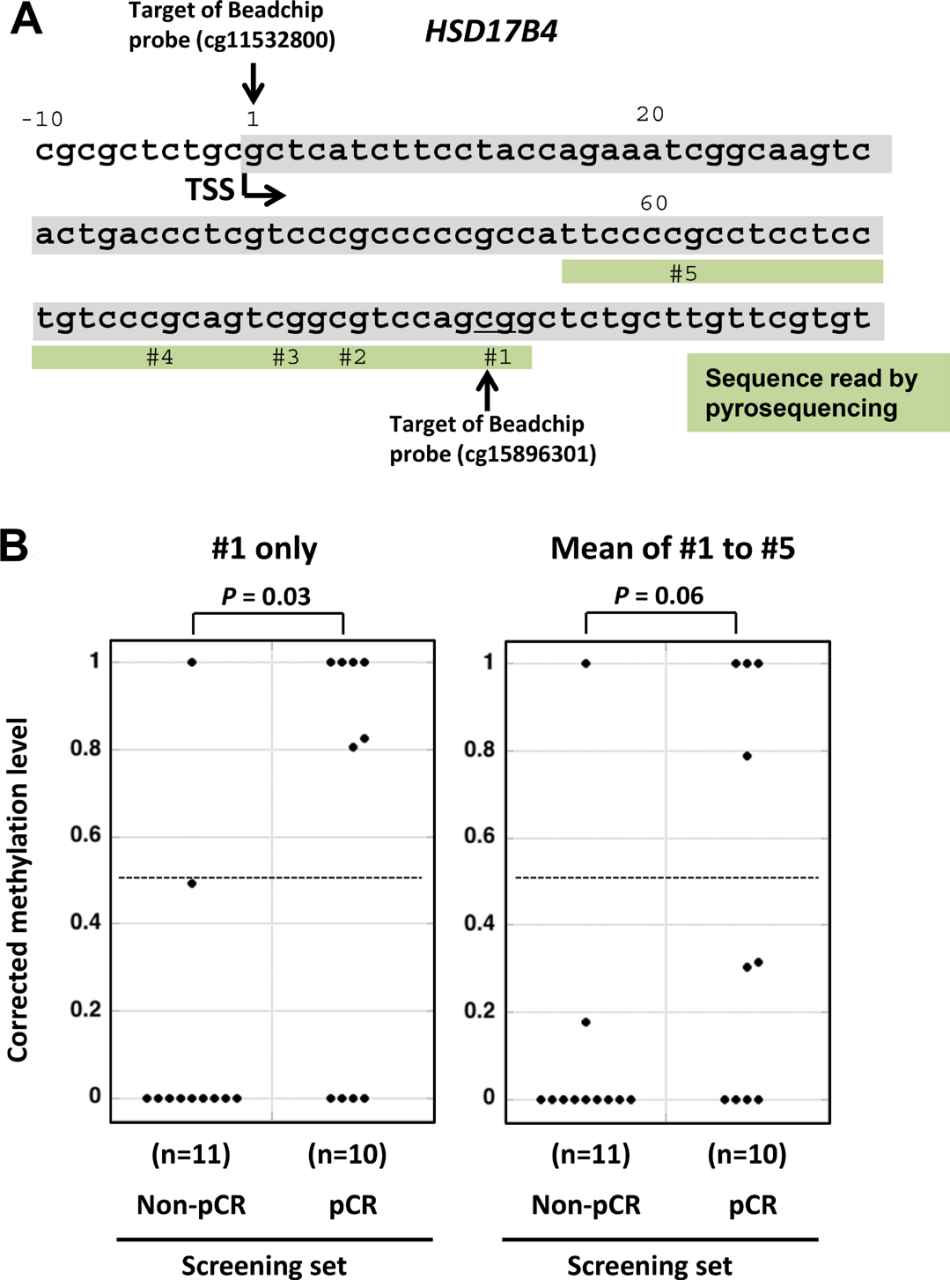
Confirmation of the differential *HSD17B4* methylation in the screening set by pyrosequencing (**A**) Genomic sequence of *HSD17B4* and the genomic region of *HSD17B4* analyzed by pyrosequencing are shown. TSS, transcription start site. (**B**) Corrected methylation levels of the candidate genomic region in the screening set. Methylation levels of the five CpG sites (#1–#5) were measured by pyrosequencing in 10 pCR samples and 11 non-pCR samples, and were corrected by the cancer cell fraction of an individual sample. Corrected methylation levels are shown for CpG #1 (left side) and the mean of five CpG sites (right side). The corrected methylation level of CpG #1 was significantly higher in responders (*P* = 0.03). The dotted line shows the cutoff value of 0.5 optimized for this screening set and used for validation.

Since a sufficient number of (eight) candidate genomic regions were isolated, we increased the threshold of specificity to 50 %, which three regions (*HSD17B4*, *PRKX*, and *RBBP7*) still met. Further, we focused on methylation changes in 200 bp upstream and downstream of a transcription start site (TSS), which is known to be critical for gene silencing [17], and *HSD17B4* and *RBBP7* remained as strong candidates. To confirm differential methylation of these two genes, we attempted to design pyrosequencing primers. While it was impossible to design a pyrosequencing primer for *RBBP7*, we successfully designed a primer for five CpG sites of *HSD17B4* (Figure 2A).

The *HSD17B4* methylation levels were measured by pyrosequencing of the 21 samples in the screening set, and, to exclude the effect of contaminating non-cancerous cells in a sample, a corrected methylation level was calculated using the ratio of cancer cells to total cells derived from microscopic observation (cancer cell fraction). The corrected methylation level of one *HSD17B4* CpG site was shown to be significantly higher in pCR samples than in non-pCR samples (*P* = 0.03; Figure 2B, left). The corrected mean methylation level of the five CpG sites tended to be higher in the pCR samples than in the non-pCR samples, but the difference was not significant (*P* = 0.06; Figure 2B, right). Therefore, we decided to use the CpG position #1 of *HSD17B4*, and a cutoff value of 0.5 for responders was established so that the Youden index (sensitivity + specificity − 1) would be maximized.

### Validation of the association between *HSD17B4* methylation and pCR to trastuzumab and chemotherapy

**Figure 3:**
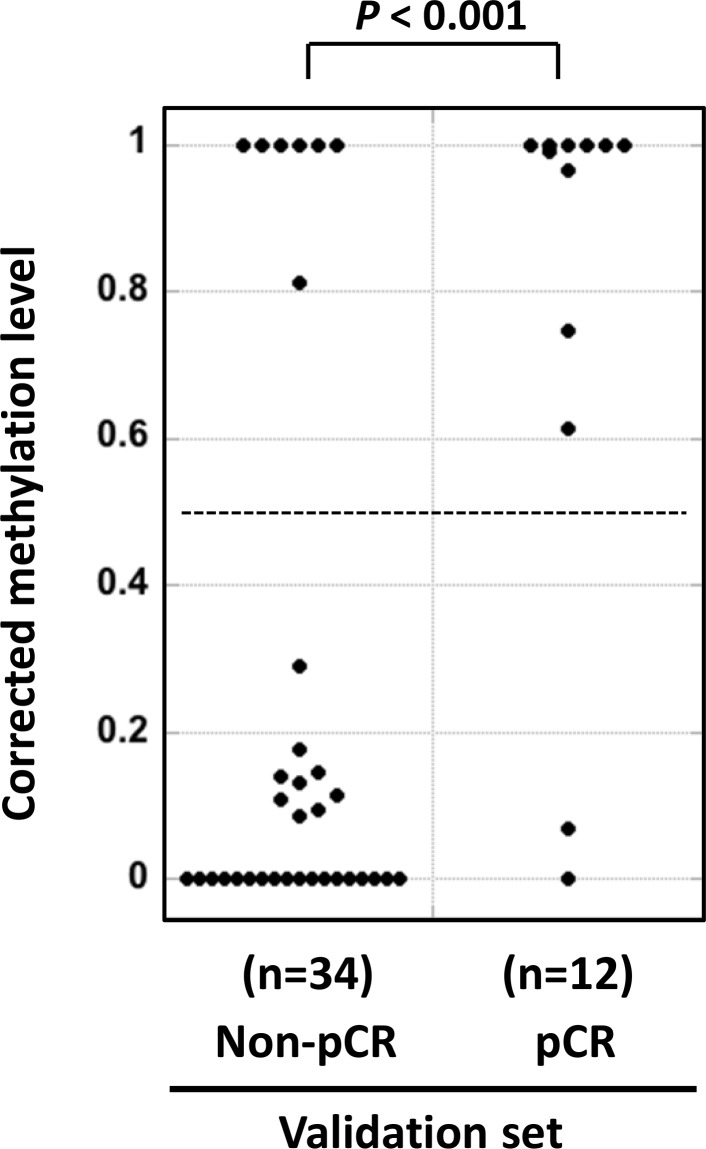
Successful validation of the differential methylation of *HSD17B4* Methylation levels of *HSD17B4* were measured by pyrosequencing in 12 pCR samples and 34 non-pCR samples in the validation set using the same methodology (pyrosequencing) and threshold established in the screening set. pCR samples had significantly higher corrected methylation levels (*P* < 0.001), and they were predicted with a sensitivity of 83 % and specificity of 79 %. The horizontal dotted line shows the cutoff value of 0.5, which had been prefixed in the screening set.

To validate the association between *HSD17B4* methylation and pCR to trastuzumab and chemotherapy, the methylation level of CpG site #1 of *HSD17B4* was analyzed in an independent sample set (validation set, Table 1), and was then corrected by the cancer cell fraction in a sample. The corrected methylation level of *HSD17B4* was found to be significantly higher in pCR samples than in non-pCR samples (*P* < 0.001; Figure 3). Using the cutoff value of 0.5, which had been prefixed in the screening set, the incidence of *HSD17B4* methylation in responders was significantly higher than in non-responders (*P* = 0.0002). These results established an association between *HSD17B4* methylation and the pCR to trastuzumab and chemotherapy. pCR to trastuzumab and chemotherapy was predicted with a sensitivity of 83%, specificity of 79%, and a positive predictive value of 53%.

### Association between *HSD17B4* methylation and clinicopathological characteristics

The predictive power of *HSD17B4* methylation was examined in comparison with clinicopathological factors. Univariate analyses revealed that ER status and *HSD17B4* methylation were significantly associated with pCR to trastuzumab and chemotherapy (Table 3). A multivariate analysis using all clinicopathological factors, including cT, cN, cStage, ER status, PgR status, and Ki-67 labelling index, showed that *HSD17B4* methylation was an independent predictive factor for pCR to trastuzumab and chemotherapy (OR 10.0, 95% CI 2.54–39.50, *P* = 0.001) (Table 4).

**Table 3:** Association between *HSD17B4* methylation and clinicopathological characteristics

Factors	*HSD17B4*		*P* value^a^
	Methylated(*N* = 24)	Unmethylated(*N* = 43)	
Clinical tumor stage			
cT1	1	3	0.76
cT2	19	30	
cT3	3	8	
cT4	1	2	
Clinical nodal stage			
cN0	9	16	1
cN1	12	21	
cN2	1	4	
cN3	2	2	
Clinical stage			
IIA	9	18	1
IIB	9	13	
IIIA	3	8	
IIIB	1	2	
IIIC	2	2	
Histology			
Invasive ductal carcinoma	23	41	1
Others	1	2	
ER^b^ status			
Positive	7	27	0.01
Negative	17	16	
PgR^c^ status			
Positive	5	21	0.04
Negative	19	22	
Ki-67 labelling index (%)			
50 ≥	15	18	0.13
50 <	9	25	
pCRd			
Positive	16	6	2.20E-05
Negative	8	37	

^a^*P* values were calculated by Fisher's exact test

^b^Estrogen receptor,^c^Progesterone receptor

^d^Pathological complete response

**Table 4:** Multivariate logistic regression analysis to identify independent factors to determine the response to trastuzumab and chemotherapy

Factors	No. of patients	Odds ratio	95% CI	*P* value
*HSD17B4* (methylated vs ummethylated)	24/43	10.02	2.54–39.50	0.001
cT (cT3 + cT4 vs cT1 + cT2)	14/53	0.55	0.03–9.32	0.681
cN (cN1 + cN2 + cN3 vs cN0 )	42/25	1.79	0.38–8.48	0.463
cStage (III vs II)	18/49	1.28	0.09–18.61	0.858
ER status (negative vs positive)	33/34	6.65	0.75–59.32	0.090
PgR status (negative vs positive)	41/26	0.84	0.07–9.61	0.890
Ki67 labelling index (< 50 vs ≥ 50)	34/33	0.46	0.11–1.92	0.285

To further increase the specificity, we explored combining *HSD17B4* methylation with ER status. ER status has been known to be associated with pCR in HER2-positive breast cancer [18], but the above multivariate analysis showed that *HSD17B4* methylation provides information independent from the ER status. The independence was confirmed by stratifying the samples into ER+ and ER- tumors, and observing significant associations in both groups (Fisher's exact test; *P* = 0.048 for ER+ tumors, and *P* = 0.005 for ER- tumors) (Supplementary Tables 2 and 3). When a sample with *HSD17B4* methylation and negative ER was scored positive, pCR to trastuzumab and chemotherapy was predicted with a sensitivity of 59%, specificity of 91%, and a positive predictive value of 76% (13/17).

## DISCUSSION

As a marker to predict pCR to trastuzumab and chemotherapy against HER2-positive breast cancer, *HSD17B4* methylation was successfully identified by genome-wide methylation analysis, and was validated by analysis of an independent validation set of samples (*P* < 0.001). This was achieved by securing the samples for validation in advance, and enabled us to exclude the concern of over-fitting. The sensitivity and specificity in the validation set were 83% and 79%, respectively, after applying the cutoff value established in the screening set. Multivariate analysis showed that *HSD17B4* methylation was an independent predictive factor for pCR. These results therefore show that a patient with *HSD17B4* methylation is highly probable to achieve pCR with trastuzumab and chemotherapy of a primary HER2-positive breast cancer.

When combined with negative ER status, a high specificity of 91% was achieved with a reasonable sensitivity of 59 %. This shows that HER2-positive/ER-negative breast cancer patients with *HSD17B4* methylation are highly likely to achieve pCR. Since pCR is associated with excellent disease-free survival in HER2-positive breast cancer patients with HER2-directed therapy [11, 12], this group of patients might not need surgery. Considering this potential application to surgery-free treatment and serious results due to false-positive diagnosis, we placed emphasis on high specificity, rather than high sensitivity. In addition, to further improve the specificity, we are trying to introduce a molecular, not pathological, marker for cancer cell fractions. The use of such a marker will enable us to assess *HSD17B4* methylation levels and cancer cell fractions in the same DNA samples, and accuracy of methylation levels in cancer cells will increase. Even after further improvement of specificity, the usefulness of *HSD17B4* methylation should be confirmed by a prospective clinical study with a sufficiently large sample size, and the study must be designed with minimum risk for the participants.

Methylation of the *HSD17B4* region, located immediately downstream of the transcription start site and within a CpG island, was associated with its silencing (Supplementary Figure 1), which was in line with a previous study that showed *HSD17B4* can be methylation-silenced [19]. However, it remains an issue how *HSD17B4* silencing is associated with pCR of HER2-positive breast cancer patients after trastuzumab and chemotherapy. *HSD17B4* encodes type 4 17β-hydroxysteroid dehydrogenase. First, this enzyme is involved in the conversion of active 17β-estradiol into inactive estrone [20]. *HSD17B4* is specifically down-regulated in breast and ovarian cancers among various human cancers [21], and this suggests that its silencing is associated with sex hormone metabolism and then sensitivity to trastuzumab. Alternatively, the enzyme is involved in β-oxidation of fatty acids and alcohols [22]. In addition, *HSD17B4* overexpression has been reported to be associated with aggressive phenotypes in liver and prostate cancers [23, 24]. This suggests that *HSD17B4* silencing is associated with sensitivity to trastuzumab via accumulation of a specific fatty acid or other metabolite and might not be related to estrogen metabolism. Further extensive analyses are necessary to clarify the underlying molecular mechanism.

In addition to the potential application to surgery-free treatment of HER2-positive breast cancer patients, unmethylated *HSD17B4* may be used to identify patients who will not benefit from trastuzumab treatment but might benefit from lapatinib. This drug is currently used only as a secondary treatment for breast cancer that has progressed after treatment with trastuzumab [25]. Naturally, again, studies with a sufficiently large number of samples from patients who responded to lapatinib and those who did not is essential to explore this important possibility.

Methodologically, CpG site #1 showed better discrimination than the mean of CpG sites #1 to #5. It is known that CpG sites closer to a sequencing primer yield more accurate values than those further away [26]. The accurate measurement of the methylation level of CpG site #1, which is closest to the pyrosequencing primer (Figure 2A), was considered to be the major reason for its good performance. To support this interpretation, the neighboring probe of the bead array (cg11532800) showed a statistically significant difference in the methylation level between the responders and non-responders, as shown in Supplementary Table 1.

In summary, we identified that methylation of the promoter CpG island of *HSD17B4* was associated with the pCR of HER2-positive breast cancer to trastuzumab and chemotherapy with a specificity of 79%. When combined with negative ER status, *HSD17B4* methylation had a high specificity of 91 %. This high specificity may pave the way for selecting HER2-positive breast cancer patients who would achieve pCR only by HER2-directed therapy and might not need additional surgery, namely surgery-free treatment of breast cancer.

## MATERIALS AND METHODS

### Tissue samples

A total of 67 tissue samples were collected from the patients enrolled in a neoadjuvant clinical trial, which will be reported elsewhere (Table 1). All the patients in this study were treated with trastuzumab and chemotherapy involving paclitaxel or anthracycline, according to the Japanese guideline of breast cancer treatment [27]. Briefly, the trial aims are: i) to explore a predictive marker of response against HER2-directed therapy using trastuzumab and chemotherapy involving paclitaxel or anthracycline, and ii) to determine whether the clinical response can be optimized by assessing a change in the Ki-67 index during neoadjuvant therapy. For the latter aim, the trial had two arms, but all samples for this study were obtained from patients who received weekly paclitaxel (80 mg/m^2^ a week) and trastuzumab (a loading dose of 4 mg/kg followed by 2 g/kg a week) for a total of 12 doses. The patients also underwent partial or simple mastectomy, and the response was pathologically assessed. The study protocol was approved by the National Cancer Center Ethics Committee (approval no. 2010–250), and was registered at the UMIN Clinical Trial Registry (Registration no. UMIN000007074) [28]. All patients provided written informed consent.

Two specimens were obtained from each patient by core needle biopsy of a primary tumor before starting neoadjuvant therapy, and fixed using two different methods. One specimen was fixed with 10% neutral buffered formalin for microscopic examination using thin sections stained with H&E, while the other was fixed using the PAXgene Tissue System (Qiagen, Hilden, Germany) and embedded in low-melting paraffin for DNA/RNA extraction using 10 slices of 10-μm sections. A certificated and experienced pathologist (S. F.) conducted the microscopic examination of biopsy specimens to determine the fraction of cancer cells and to select specimens containing a sufficient number of tumor cells for molecular analyses. The pathologist also analyzed surgical specimens to determine the therapeutic response. pCR was defined as no residual tumor cells in surgical specimens.

The 67 HER2-positive breast cancer samples were histologically classified as 64 invasive ductal carcinomas, two invasive lobular carcinomas, and one other histological type according to WHO classification. Thirty-four (50.7%) breast cancer samples were positive for estrogen receptor (ER), and 26 (38.8%) were positive for progesterone receptor (PgR). The samples were divided into a screening set (*n* = 21) and a validation set (*n* = 46). Only samples with a tumor cell fraction of 40% or more were used.

### Genome-wide DNA methylation analysis

Genome-wide DNA methylation analysis was conducted using an Infinium HumanMethylation450 BeadChip array (Illumina, San Diego, CA), which assessed the degree of methylation of 485,512 probes (482,421 CpG sites and 3,091 non-CpG sites). The methylation level of each probe was obtained as a β value, which ranged from 0 (completely unmethylated) to 1 (completely methylated). Intra-array normalization was performed to exclude probe design biases using the peak-based correction method Beta Mixture Quantile dilation [29]. A corrected β value was obtained from the normalized β value based on the fraction of cancer cells in the sample [corrected β value = 100 × (normalized β value) / (fraction of cancer cells in the sample {%})].

### Bisulfite pyrosequencing

Sodium bisulfite modification was performed using 1 μg of genomic DNA as previously described [30]. The modified DNA was suspended in 40 μl of Tris–EDTA buffer, and an aliquot of 2 μl was used for bisulfite pyrosequencing. All pyrosequencing primers were obtained as PyroMark CpG Assays for methylation array validation (Qiagen, Valencia, CA). PCR products labelled with biotin were annealed to 0.2 μM pyrosequencing primers, and pyrosequencing was carried out using the PSQ 96 Pyrosequencing System (Qiagen). Methylation levels were obtained using PSQ Assay Design software (Qiagen).

The measured methylation level was corrected according to the fraction of cancer cells in a sample [corrected methylation level = 100 × (measured methylation level) / (fraction of cancer cells in the sample {%})]. The fraction of cancer cells (%) was obtained by microscopic observation.

### Statistical analysis

Fisher's exact test was used to evaluate the significant difference in relative frequency between two groups. Differences in normalized methylation levels between the responders and non-responders were evaluated by the Mann–Whitney *U* test. In the univariate analysis, odds ratios (ORs) and 95% confidence intervals (95% CIs) were calculated. Multivariate logistic regression analyses and other statistical analyses were conducted using PASW statistics version 18.0.0 (SPSS Japan Inc., Tokyo, Japan).

## SUPPLEMENTARY MATERIALS FIGURES AND TABLES



## Abbreviations

HER2: human epidermal growth factor 2
pCR: pathological complete response
HSD17B4: type 4 17β-hydroxysteroid dehydrogenase
H&E: hematoxylin and eosin
OR: odds ratio
CI: confidence interval
ER: estrogen receptor
PgR: progesterone receptor.
