# Supplementary material for: Pathological complete response of HER2-positive breast cancer to trastuzumab and chemotherapy can be predicted by HSD17B4 methylation
Source: Oncotarget. 2017 Feb 6;8(12):19039–48. doi: 10.18632/oncotarget.15118 (PMC5386667; doi:10.18632/oncotarget.15118)
Supplement: Supplementary file 1 [file oncotarget-08-19039-s001.pdf]

Pathological complete response of HER2-positive breast cancer to trastuzumab and chemotherapy can be predicted by *HSD17B4* methylation

Supplementary Materials

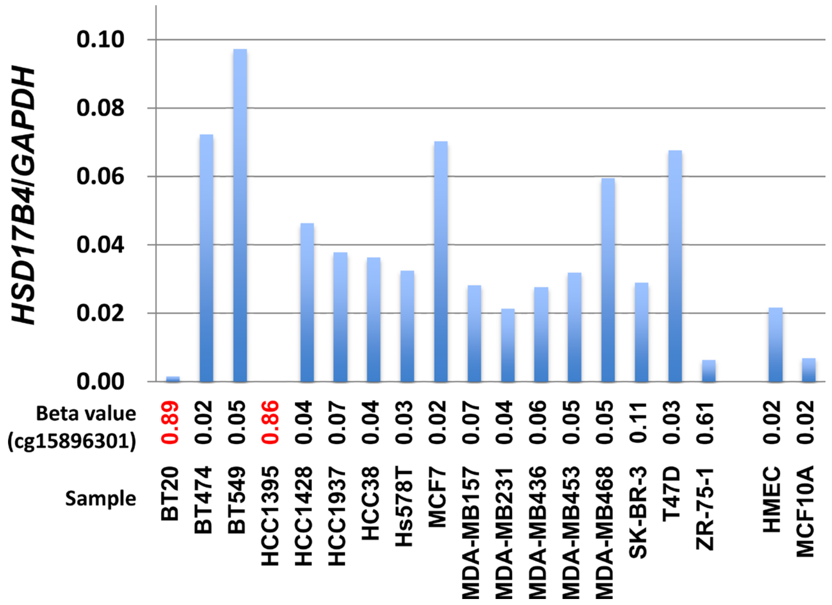

Supplementary Figure 1: *HSD17B4* mRNA expression levels in breast cancer cell lines and breast epithelial cells were analyzed by real-time RT-PCR. *HSD17B4* was not expressed in two breast cancer cell lines with high methylation (BT20 and HCC1395).

Supplementary Table 1: Probes in the eight genomic regions identified by genome-wide DNA methylation analysis. See Supplementary_Table _1

Supplementary Table 2: Relationship between *HSD17B4* methylation and pCR in ER-positive tumors

	***HSD17B4* methylation (+)**	***HSD17B4* methylation (–)**
pCR	3	2
non-pCR	4	25

Fisher's exact test, *P* = 0.048.

Supplementary Table 3: Relationship between *HSD17B4* methylation and pCR in ER-negative tumors

	**HSD17B4 methylation (+)**	**HSD17B4 methylation (–)**
pCR	13	4
non-pCR	4	12

Fisher's exact test, *P* = 0.005.
